# ZAP-X Stereotactic Radiosurgery for Optic Nerve Sheath Meningioma: A Case Report

**DOI:** 10.7759/cureus.87094

**Published:** 2025-07-01

**Authors:** Michael Chaga, Timothy Chen, Wenzheng Feng, Patrick Pema, Harshal Shah, Tingyu Wang, Darra Conti, Jing Feng, Ma Rhudelyn Rodrigo, Elizabeth Luick, Daniel Thompson, Joy Baldwin, Brielle Latif, Joseph Hanley, Shabbar Danish

**Affiliations:** 1 Radiation Oncology, Jersey Shore University Medical Center, Neptune, USA; 2 Neurosurgery, Hackensack Meridian School of Medicine, Nutley, USA; 3 Neurosurgery Surgery, Jersey Shore University Medical Center, Neptune, USA

**Keywords:** gyroscopic, gyroscopic radiosurgery, gyroscopic stereotactic radiosurgery, onsm, optic nerve sheath meningioma, radiosurgery, srs, stereotactic radiosurgery, zap-x

## Abstract

Primary optic nerve sheath meningiomas (ONSMs) are benign tumors and are a common cause of optic neuropathy due to compression of the optic nerve. Management of this tumor can be surgery or radiotherapy, but radiotherapy is the preferred treatment modality due to the risk of damage to the vasculature and resulting vision loss in surgery. The ZAP-X (ZAP Surgical Systems, Inc., San Carlos, CA) is currently the newest cranial stereotactic radiosurgery (SRS) platform. There are no reports describing its use or outcomes for ONSM. We present the case of the first documented patient to undergo ZAP-X SRS for the treatment of a primary ONSM.

The patient was a 68-year-old woman with a 0.27 cm^3^ enhancing lesion centered along the left optic nerve near the orbital apex with unilateral fluid distention of the left optic nerve sheath. The treatment plan consisted of six isocenters placed in the target and with a prescription dose of 2500 cGy in five fractions at the 61% isodose line. The maximum dose to 0.035 cm^3^ (D0.035cc) of the left and right optic nerves was 1454.9 and 26.4 cGy, respectively. Conformity and gradient indexes were 1.638 and 3.451, respectively.

The patient tolerated the procedure well with no complications. Six months post-treatment, the patient had completely resolved left eye pain and pressure, with significantly reduced proptosis and a 44% reduction in tumor volume with no vasogenic edema. This case presents the successful use of ZAP-X SRS for the treatment of ONSM. Although requiring further investigation, such studies are needed to define the long-term efficacy of the platform.

## Introduction

Primary optic nerve sheath meningiomas (ONSMs) are benign tumors that account for approximately 2% of all orbital tumors and are a common cause of optic neuropathy due to compression of the optic nerve. The presentation of this tumor is variable but can consist of vision loss, visual field defects, and proptosis. Management of this tumor can be surgery or radiotherapy, but radiotherapy is the preferred treatment modality due to the risk of damage to the vasculature and resulting vision loss in surgery [1,2]. 

The ZAP-X (ZAP Surgical Systems, Inc., San Carlos, CA) is the first clinical self-shielded stereotactic radiosurgery (SRS) platform and consists of a 3 MV Linear Accelerator (Linac) mounted on axial and oblique gimbals, allowing for isocentric, non-coplanar, dual-axis radiation delivery over a solid angle of greater than 2π steradians. The unique shielding design of the ZAP-X reduces radiation exposure levels inside the treatment room, eliminating the need for a shielded vault while increasing installation flexibility [3-5]. Compared to other common radiotherapy methods of Gamma Knife and CyberKnife, the ZAP-X produced the lowest full-width half maximum and sharpest overall penumbra for its smallest collimator [6]. The ZAP-X is currently the newest cranial SRS platform. We present the case of the first documented patient to undergo ZAP-X SRS for the treatment of a primary ONSM.

## Case presentation

The patient was a 68-year-old woman diagnosed with an ONSM who presented with proptosis. The patient had lost vision in her left eye 10 years prior, the cause of which was diagnosed as macular degeneration. The patient first noticed proptosis six months prior to presentation; this finding occurred in the setting of near complete blindness with only minor light perception in the same eye.

**Figure 1:**
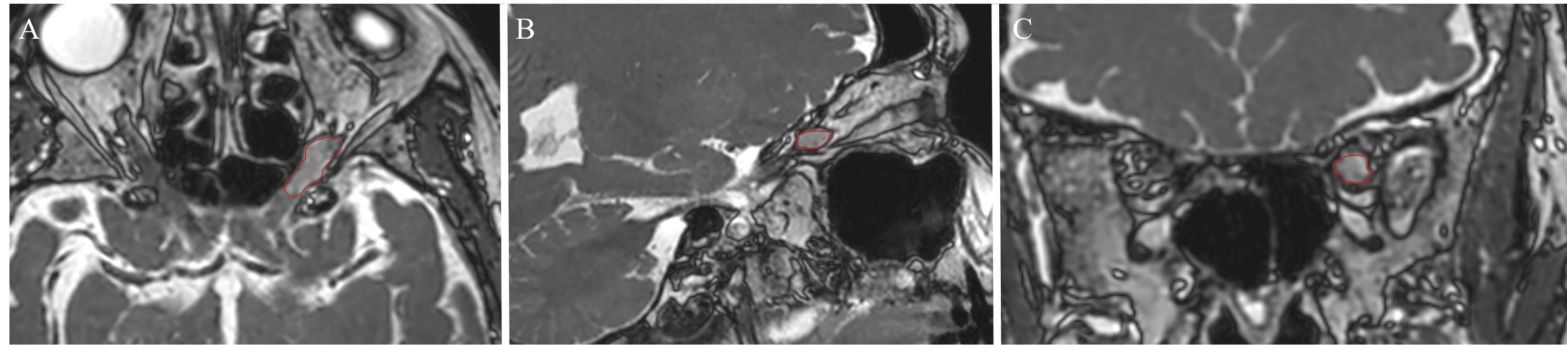
Pre-treatment SSFP MRI: A) axial section, B) sagittal section, and C) coronal section (ONSM shown in red). The tumor displayed an increased fluid signal in the left optic nerve sheath with edema. Image Credit: Michael Chaga SSFP: steady-state free precession; ONSM: optic nerve sheath meningioma

Magnetic resonance imaging (MRI) revealed a 0.27 cm^3^ enhancing lesion centered along the left optic nerve near the orbital apex with unilateral fluid distention of the left optic nerve sheath (Figure 1). Radiology and Neurosurgery determined that this finding was related to cerebrospinal fluid outflow obstruction. After referral to and visitation with Radiation Oncology and Neurosurgery, those departments, along with the patient, agreed upon treatment with the ZAP-X platform. Although it was noted that the patient was unlikely to regain vision, this option was chosen with the goals of preventing further tumor growth and alleviating proptosis symptoms.

**Figure 2:**
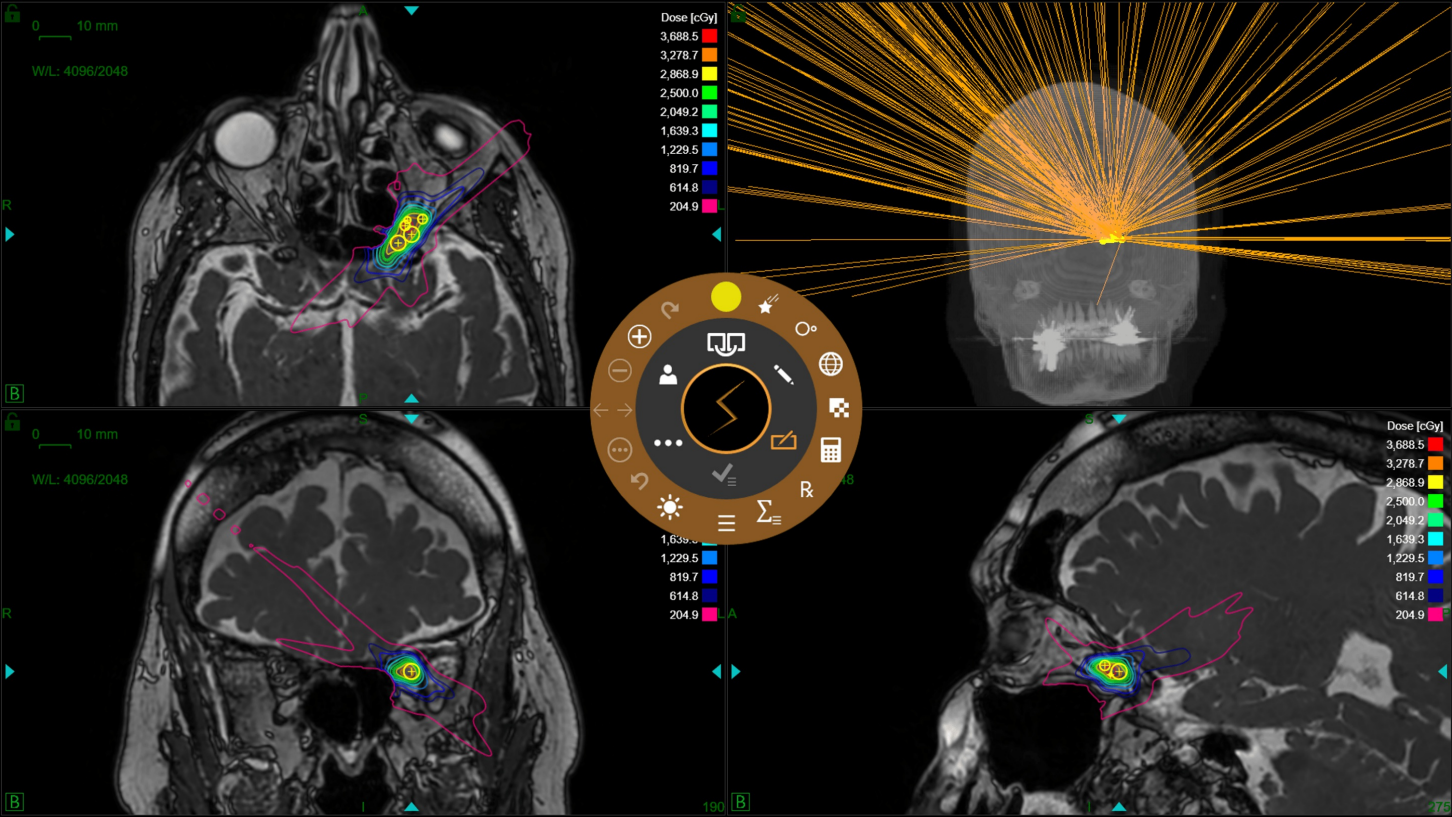
ZAP-X ONSM SRS with six isocenters placed in the target and a prescription dose of 2500 cGy in five fractions at the 61% isodose line. Image Credit: Michael Chaga ONSM: optic nerve sheath meningioma; SRS: stereotactic radiosurgery

For treatment planning, computerized tomography (CT) simulation was performed on a GE Optima CT660. CT simulation images were acquired with 0.625 mm slice thickness and 0.488 × 0.488 mm in-plane resolution. MRI simulation was performed with gadolinium contrast on a 0.5 T Synaptive MRI consisting of a steady-state free precession (SSFP) sequence with 0.8 mm resolution, matrix size 300 × 216 × 224, repetition time 6.78 ms, and flip angle 35°. Volumes of interest were contoured by a qualified Radiation Oncologist, Neurosurgeon, and Medical Physicist utilizing a fused dataset composed of the treatment planning CT and post-gadolinium MRI images within the Eclipse (version 15.6) (Varian Medical Systems, Palo Alto, CA) treatment planning system (TPS). There was no margin applied to the target volume. The contoured images were then imported to the ZAP-X TPS (version 1.8.58). The treatment plan consisted of six isocenters placed in the target and with a prescription dose of 2500 cGy in five fractions at the 61% isodose line. The plan utilized 4 and 5 mm collimators, path 10 gantry movement, and 252 beams. Forward- and inverse-planning was performed, where isocenters were manually placed first, followed by inverse optimization of beam weights and manual addition, deletion, or repositioning of isocenters. A 0.5 mm dose grid was utilized, limiting the dose to the eyes, lens, optic nerves, optic chiasm, cochleae, brainstem, and spinal cord based on Timmerman organ-at-risk (OAR) recommendations [7]. Plan quality was evaluated for conformality and dose fall-off using the conformity index (CI) and gradient index (GI). The definition for the CI utilized was the ratio of the prescription isodose volume and the target volume [8]. The definition for the GI utilized was the ratio of half the prescription isodose volume and the prescription isodose volume [9]. Radiation Therapy Oncology Group Protocol 0813 was utilized for plan quality constraints [10]. The ZAP-X treatment plan is demonstrated in Figure 2.

For quality assurance (QA), an independent second monitor unit (MU) check was performed with an in-house developed program supported for CyberKnife utilizing ZAP-X beam data including cone factors, tissue phantom ratios, and off-center ratios with a tolerance of ≥ 95% maximum dose accuracy [11]. Patient-specific QA was performed using SRS MapCHECK and gamma analysis was performed using the SunCHECK Patient software (Sun Nuclear, Melbourne, FL) with 6FFF angular correction, low-dose threshold 10%, distance-to-agreement 1%, dose difference 2%, and gamma passing rate criteria ≥ 95%. Prior to each treatment fraction, recommended daily QA tests were performed utilizing functional, Winston-Lutz (WL), and output tests. Average WL x, y, and z-offsets were 0.11 ± 0.02, 0.42 ± 0.03, and 0.10 ± 0.02 mm, respectively. Maximum WL x, y, and z-offsets were 0.15, 0.45, and 0.13 mm, respectively.

To ensure patient comfort and positioning, the patient was placed in mold care and a thermoplastic Fibreplast mask (CQ Medical, Avondale, PA). The patient alignment process involves a sequence of 3D alignment steps using non-coaxial kV x-ray images from multiple gantry angles. The images are co-registered to digitally reconstructed radiographs generated from the initial CT used in the treatment plan. The initial auto-alignment before treatment was approved by a Radiation Oncologist. Alignment deviation of more than 2 mm in any direction and 1.5° in any rotation axis would require a readjustment of the patient positioning and repeating auto-alignment and Radiation Oncologist approval. If > 5 gantry locations with MV dosimetry deviation of more than 10% exist, physicist investigation is required. Treatment would be stopped for further investigation with the vendor if > 10 gantry locations with MV dosimetry deviation of more than 10% exist.

Table 1 summarizes the various dosimetric parameters. Table 2 summarizes plan quality metrics and doses to the target and OARs. All machine QA tests were within the recommended tolerances. Secondary check maximum dose accuracy was 99.7%. The gamma passing rate was 99%. The average treatment time per fraction was 38.2 ± 1.6 minutes. The maximum dose to 0.035 cm^3^ (D0.035cc) of the left and right optic nerves was 1454.9 and 26.4 cGy, respectively. The volume receiving 2300 cGy (V2300cGy) for the left and right optic nerves was 0.006 and 0 cm^3^, respectively. The maximum point dose (D_max_) to the left and right eye was 82.1 and 22.2 cGy, respectively. D_max_ to the left and right lens was 54.1 and 19.2 cGy, respectively. Optic chiasm D0.035cc and V2300cGy were 515.7 cGy and 0.001 cm^3^, respectively. The CI and GI were 1.638 and 3.451, respectively.

**Table 1:** ZAP-X ONSM SRS dosimetric results delivering 2500 cGy in five fractions. Average ± standard deviation (range). Target sphericity calculated with the Radiomics extension of 3D-Slicer software. Setup Time is auto-alignment imaging and review duration. Gantry Time is the duration for the gantry to move all gantry angles throughout the entire delivery. Table Time is the duration for the table to move to all table locations throughout the entire delivery. kV Imaging and Processing Time is the duration to acquire the kV images throughout the entire delivery, match to DRRs, and perform noise reduction, edge detection, and normalization. Linac Time is the beam on delivery duration.

Dosimetric Parameter	ZAP-X ONSM SRS
Target Volume (cm^3^)	0.27
Target Sphericity	0.626
Prescription Dose (cGy)	2500
Prescription Isodose Line (%)	61
Fraction Number	5
Number of Isocenters	6
Number of Beams	252
Second MU Check Maximum Dose Accuracy (%)	99.7
Gamma Passing Rate (%)	99
WL x-offset (mm)	0.11 ± 0.02 (0.09 – 0.15)
WL y-offset (mm)	0.42 ± 0.03 (0.37 – 0.45)
WL z-offset (mm)	0.10 ± 0.02 (0.08 – 0.13)
Delivered MU/Fx	7423.75 ± 0.13 (7423.54 – 7423.87)
Treatment Time (min/Fx)	38.2 ± 1.6 (36.6 – 40.6)
Setup Time (min/Fx)	3.6 ± 1.7 (2.3 – 6.4)
Gantry Time (min/Fx)	21.3 ± 1.2 (20.6 – 23.3)
Table Time (min/Fx)	0.464 ± 0.013 (0.45 – 0.48)
kV Imaging and Processing Time (min/Fx)	7.6 ± 1.5 (5.2 – 9.2)
Linac Time (min/Fx)	5.27 ± 0.03 (5.23 – 5.3)

**Table 2:** ZAP-X ONSM SRS plan quality metrics and doses to the target and OARs. GTV: gross tumor volume; V100%: prescription GTV coverage; CI: conformity index; GI: gradient index; D0.035cc: maximum dose to 0.035 cm^3^; V2300cGy: volume receiving 2300 cGy; D_max_: maximum point dose; V2200cGy: volume receiving 2200 cGy; V3650cGy: volume receiving 3650 cGy; V2400cGy: volume receiving 2400 cGy; OAR: organ-at-risk; SRS: stereotactic radiosurgery

Dosimetric Parameter	Metric	Constraint	ZAP-X ONSM SRS
GTV	V100% (%)	≥ 95%	98.69%
	CI	≤ 1.2 – 1.5	1.638
	GI	≤ 4.3 – 5	3.451
Left Optic Nerve	D0.035cc (cGy)	≤ 2500 cGy	1454.9 cGy
	V2300cGy (cm^3^)	≤ 0.2 cm^3^	0.006 cm^3^
Right Optic Nerve	D0.035cc (cGy)	≤ 2500 cGy	26.4 cGy
	V2300cGy (cm^3^)	≤ 0.2 cm^3^	0 cm^3^
Optic Chiasm	D0.035cc (cGy)	≤ 2500 cGy	515.7 cGy
	V2300cGy (cm^3^)	≤ 0.2 cm^3^	0.001 cm^3^
Left Cochlea	D0.035cc (cGy)	≤ 2200 cGy	61.2 cGy
Right Cochlea	D0.035cc (cGy)	≤ 2200 cGy	23.3 cGy
Left Lens	D_max_ (cGy)	≤ 100 – 200 cGy	54.1 cGy
Right Lens	D_max_ (cGy)	≤ 100 – 200 cGy	19.2 cGy
Left Eye	D_max_ (cGy)	≤ 100 – 200 cGy	82.1 cGy
Right Eye	D_max_ (cGy)	≤ 100 – 200 cGy	22.2 cGy
Brainstem (excluding medulla)	D0.035cc (cGy)	≤ 3100 cGy	108.5 cGy
	V2300cGy (cm^3^)	≤ 0.5 cm^3^	0 cm^3^
Spinal Cord (including medulla)	D0.035cc (cGy)	≤ 2800 cGy	24.4 cGy
	V2200cGy (cm^3^)	≤ 0.35 cm^3^	0 cm^3^
Skin	D0.035cc (cGy)	≤ 3850 cGy	404.1 cGy
	V3650cGy (cm^3^)	≤ 10 cm^3^	0 cm^3^
Brain - GTV	V2400cGy (cm^3^)	≤ 16.8 cm^3^	0.001 cm^3^

**Figure 3:**
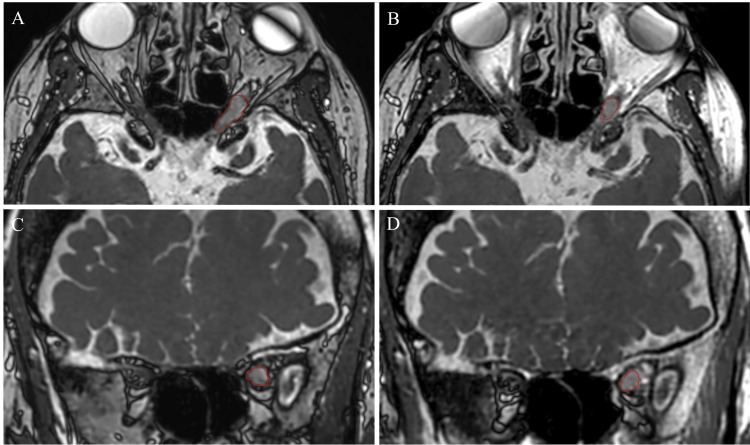
Comparison of pre- and post-treatment SSFP MRIs at six-month follow-up: A) pre-treatment axial section, B) post-treatment axial section, C) pre-treatment coronal section, and D) post-treatment coronal section. At six months post-treatment, there was a 44% reduction in the tumor volume. Image Credit: Michael Chaga SSFP: steady-state free precession

The patient tolerated the procedure well with no complications. Six months post-treatment, the patient had completely resolved left eye pain and pressure, with significantly reduced proptosis. Upon follow-up MRI imaging (Figure 3), the tumor volume was contoured and measured to be 0.15 cm^3^, a 44% reduction in tumor volume with no vasogenic edema. However, there was no improvement in the patient’s vision, and the patient continues to only have light perception in the left eye.

## Discussion

ONSMs account for about one-third of primary optic nerve tumors. Vision loss is the most common clinical manifestation of an ONSM, and while the degree of visual loss varies highly by patient, this symptom is experienced by over 90% of patients. ONSMs may cause progressive vision loss far before they are discovered on imaging [12]. Indeed, our patient exhibited progressive vision loss over the years and visited multiple doctors before being diagnosed with a meningioma, showing the subtle nature of the tumor and how it can be difficult to diagnose due to slow progression [13,14]. Thus, the manner in which the slow growth of the ONSM affected the patient’s visual symptoms is unknown. By the time the meningioma was discovered on imaging, the patient’s vision loss was not able to be recovered. It is possible that an earlier discovery and diagnosis of optic nerve sheath meningioma, followed by a course of SRS, could have slowed or eliminated the patient’s visual symptom progression. Current literature supports this theory, as SRS has been shown to improve visual symptoms in at least 50% of affected patients. Notably, visual acuity prior to treatment is a strong predictor of whether or not vision will improve after SRS [14].

Even without the possibility of restoring our patient’s vision, SRS is indicated for the goals of symptomatic improvement and slowing tumor progression in important anatomical locations. As such, tumor control is an important aim of treatment for pathologies located near the optic nerve and the optic chiasm [15]. Uncontrolled meningioma growth poses risks of both mass effect on nearby structures, such as the optic nerve [2,12]. Surgical intervention such as SRS is not indicated unless the tumor imposes such effect on the surrounding tissue. In our patient’s case, edema and pressure caused by the meningioma were confirmed. The ZAP-X therapy effectively shrunk the tumor with no complications. Therefore, while visual improvement in this patient was negligible, this treatment has important relevance for reducing mass effect on local structures. 

Proptosis, as experienced by our patient, is a common symptom of ONSM; over 50% of patients with this type of tumor may experience proptosis during the disease course [16]. This condition can cause physical discomfort, dryness, and pain, as well as an unsightly cosmetic appearance. Our patient experienced pressure, pain, and a bulging orbit and eye in this manner. Our therapy reduced proptosis completely in this patient, demonstrating its utility in resolving that specific complication. 

For ONSM SRS local tumor control and side effects, Senger et al. report a local control rate of 96%, 4% mild headache, 4% transient diplopia, with a mean follow-up of 37 months (range: 6 - 84 months) using CyberKnife (1400 - 1500 cGy at the 70% isodose line, 2000 - 2500 cGy at the 70 - 85% isodose line) [17]. Marchetti et al. report a local control rate of 100%, 10% abnormal lacrimation, 5% temporary diplopia with mild optic neuropathy, 5% dizziness, with a mean follow-up of 30 months (range: 11 - 68 months) using CyberKnife (2500 cGy at the 75 - 85% isodose line) [18]. Romanelli et al. report a local control rate of 100%, no reported toxicities, with a median follow-up of 74 months (range: 36 - 88 months) using CyberKnife (2000 cGy at the 70% isodose line) [19]. Liu et al. report a local control rate of 93.3% at five years, 13% reversible conjunctival edema, 3% transient orbital pain, 3% transient headache, with a median follow-up of 56 months (range: 38 - 108 months) using Gamma Knife (1000 - 1700 cGy) [20]. Romanelli et al. report a local control rate of 100% at three years, no reported toxicities, with a median follow-up of 32 months (range: 30 - 42 months) using CyberKnife (2000 cGy at the 80% isodose line) [21]. Using any of the definitions of local tumor control in the literature reported ONSM SRS studies, our patient would be classified as progression-free. 

The primary concern of treatment with SRS is toxicity to the surrounding tissue [22]. Thus, treatment specificity regarding contours and target volume is of the utmost importance. Our treatment with the ZAP-X system achieved optimal CI and GI indices as well as a secondary maximum dose check accuracy of 99.7%, gamma passing rate of 99%, and WL test accuracy within 0.45 mm. These values indicate high accuracy of beam delivery and safe and effective delivery to the tumor area. Our patient reported reduced symptoms and no indicators of any toxicity to the surrounding tissue. This result emphasizes the effectiveness of the ZAP-X platform in delivering accurate and nontoxic dosages to areas of high importance, such as the optic nerve and surrounding structures.

## Conclusions

This case presents the successful use of ZAP-X SRS for treating ONSM, marking the first documented case using this platform. The treatment was delivered with high precision, achieving a significant reduction in tumor volume without causing radiation-induced complications. The patient experienced a resolution of symptoms such as pain, pressure, and proptosis, highlighting the potential of ZAP-X SRS as a safe and effective option for managing ONSM. Although the patient's vision did not improve due to the advanced state of the condition prior to treatment, the primary objective of symptom alleviation and tumor volume reduction was successfully met. This outcome underscores the need for further research to evaluate the long-term efficacy and broader applicability of the ZAP-X platform in treating cranial tumors, particularly in sensitive areas like the optic nerve.
